# Supplementary material for: Hybrid Models and Biological Model Reduction with PyDSTool
Source: PLoS Comput Biol. 2012 Aug 9;8(8):e1002628. doi: 10.1371/journal.pcbi.1002628 (PMC3415397; doi:10.1371/journal.pcbi.1002628)
Supplement: Text S4 — Complete source code for the PyDSTool package (version 0.88.120504). Includes API documentation and help files linking to web pages. This file is identical to the current public release on Sourceforge.net. (ZIP) [file pcbi.1002628.s004.zip › PyDSTool/html/PyDSTool.Toolbox.optimizers.helpers.levenberg_marquardt.LMQuadratic-class.html]

xml version="1.0" encoding="ascii"?


PyDSTool.Toolbox.optimizers.helpers.levenberg\_marquardt.LMQuadratic


| Home | Trees | Indices | Help | PyDSTool |
| --- | --- | --- | --- | --- |

Package PyDSTool ::
Package Toolbox ::
Package optimizers ::
Package helpers ::
Module levenberg\_marquardt ::
Class LMQuadratic

[hide private]

[frames] | no frames]

# Class LMQuadratic

source code

```
         object --+    
                  |    
quadratic.Quadratic --+
                      |
                     LMQuadratic
```

---

Defines a cost function with a quadratic cost but the
Levenberg-Marquardt approximation of the hessian


|  |  |
| --- | --- |
| Instance Methods | [hide private] |

|  |  |
| --- | --- |
| hessian(self, params)  Compute sthe hessian of the fit function | source code |

**Inherited from `quadratic.Quadratic`**:
`__call__`,
`__init__`,
`gradient`

**Inherited from `object`**:
`__delattr__`,
`__getattribute__`,
`__hash__`,
`__new__`,
`__reduce__`,
`__reduce_ex__`,
`__repr__`,
`__setattr__`,
`__str__`


|  |  |
| --- | --- |
| Properties | [hide private] |

**Inherited from `object`**:
`__class__`


|  |  |
| --- | --- |
| Method Details | [hide private] |

|  |  |
| --- | --- |
| hessian(self, params) | source code |

Compute sthe hessian of the fit function

Overrides: quadratic.Quadratic.hessian

  


| Home | Trees | Indices | Help | PyDSTool |
| --- | --- | --- | --- | --- |

|  |  |
| --- | --- |
| Generated by Epydoc 3.0.1 on Fri May 4 15:24:09 2012 | http://epydoc.sourceforge.net |
